# The Need for a Definition of Big Data for Nursing Science: A Case Study of Disaster Preparedness

**DOI:** 10.3390/ijerph13101015

**Published:** 2016-10-17

**Authors:** Ho Ting Wong, Vico Chung Lim Chiang, Kup Sze Choi, Alice Yuen Loke

**Affiliations:** 1 Disaster Nursing Task Force, School of Nursing, the Hong Kong Polytechnic University, Hong Kong, China; frankwong@connect.hku.hk (H.T.W.); alice.yuen.loke@polyu.edu.hk (A.Y.L.); 2 Department of Geography, National Taiwan University, Taipei City 10617, Taiwan; 3 Institute of Geographic Sciences and Natural Resources Research, Chinese Academy of Sciences, Beijing 100101, China; 4 School of Nursing, the Hong Kong Polytechnic University, Hong Kong, China; thomasks.choi@polyu.edu.hk

**Keywords:** Big Data, disaster preparedness, research method

## Abstract

The rapid development of technology has made enormous volumes of data available and achievable anytime and anywhere around the world. Data scientists call this change a data era and have introduced the term “Big Data”, which has drawn the attention of nursing scholars. Nevertheless, the concept of Big Data is quite fuzzy and there is no agreement on its definition among researchers of different disciplines. Without a clear consensus on this issue, nursing scholars who are relatively new to the concept may consider Big Data to be merely a dataset of a bigger size. Having a suitable definition for nurse researchers in their context of research and practice is essential for the advancement of nursing research. In view of the need for a better understanding on what Big Data is, the aim in this paper is to explore and discuss the concept. Furthermore, an example of a Big Data research study on disaster nursing preparedness involving six million patient records is used for discussion. The example demonstrates that a Big Data analysis can be conducted from many more perspectives than would be possible in traditional sampling, and is superior to traditional sampling. Experience gained from the process of using Big Data in this study will shed light on future opportunities for conducting evidence-based nursing research to achieve competence in disaster nursing.

## 1. Introduction

Computing technology has further advanced in recent years, and the availability and popularity of social media and smart phones has led to new sources for collecting research data. These technological advancements have caused the world to evolve to an “era of data”. Enormous amounts of data can now be generated anytime and anywhere around the world [1,2]. This is certainly good news for scientists, including nursing scholars, as their research is no longer limited to conventional data collection methods.

The popularity and accessibility of social media and smart phones have led to revolutionary changes in numerous disciplines, including journalism [3,4], geography [5,6], and emergency medicine [7,8]. It is now common for journalists to search and analyze the postings on social media, resulting in a new professional area called data journalism, which emphasizes the role of data in news reports [9]. Similarly, Big Data is also facilitating research relating to disaster management among geographers. For example, the built-in Global Positioning System (GPS) in a typical smart phone is a valuable source of data. A study was conducted making use of the smart phone GPS data of 1.6 million people to develop a spatio-temporal model that simulated and predicted the moving patterns of people in Tokyo after the 11 March earthquake in 2011 [10]. Similar to smart phones, social media such as Twitter can generate tens of thousands of tweets every second around the world. The United States Geological Survey demonstrated the feasibility of monitoring the number of earthquake-related posts in tweets immediately after an earthquake to detect earthquakes in 2009. This method is particularly convenient for areas without earthquake sensors [11]. Apart from personal communication devices and online social media platforms, the government is one of the major owners of Big Data, given the tremendous amounts of data held by different public organizations. Abundant data are readily available in public hospitals regarding the health of citizens and their patterns of service usage, meaning that nursing and related research can likely benefit by utilizing such data. As revolutionary changes have already been observed in other disciplines, it is envisioned that Big Data will also have a revolutionary impact on nursing science.

**Figure 1:**
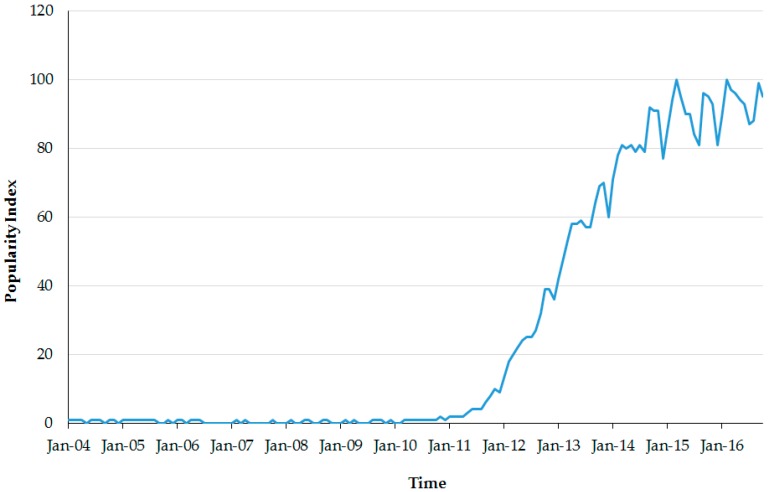
The popularity of the search query “Big Data” in Google Trends.

According to Google Trends [12], the number of Google searches using the query “Big Data” began increasing in 2011 (Figure 1). This suggests that the concept and term are becoming more popular among the public, and that the situation among nursing scholars is similar. The same search among PubMed Nursing Journals without year restrictions returned 38 publications. The first publication related to Big Data in PubMed Nursing Journals appeared in 2013 [13]. The number of such papers increased to 2 in 2013, 7 in 2014, and 19 in 2015. In 2016, a total of 10 publications were identified up to the month of June. Although the trend of increase and the total number of publications related to Big Data in PubMed Nursing Journals are not as impressive as the popularity of “Big Data” in Google searches, they show that Big Data is already having a definite impact on nursing science. Such an impact is one day expected to be revolutionary.

## 2. The Challenge of Big Data to Scholars in Nursing

Although the concept of Big Data has been attracting the attention of scholars in nursing since the birth of the era of data, the use of Big Data in nursing research remains limited. One reason for this could be the lack of a unified definition of “Big Data”. In the 38 publications in PubMed Nursing Journals (identified using the PubMed Nursing Journals full text keyword search function and the keyword “Big Data”; a full list of the 38 publications can be found in Appendix Table A1), scholars in nursing have adopted very different definitions of Big Data [14,15,16], while some made no attempts to define the term before using it [17,18]. Among the 38 publications related to Big Data in PubMed Nursing Journals, 22 explicitly defined Big Data. Nine of the 22 adopted the popular 3Vs or 4Vs definition [19], while the remaining publications adopted different, less structured, definitions. One referred to Big Data as “*any collection of data that is large and complex enough to become difficult to process*” [20]. In fact, unstandardized or imprecise definitions cause confusion to scholars, as many of them perceive Big Data as equivalent to big datasets, with a sample size “significantly bigger” than those in a “typical study”. De Mauro et al. [21] (a frequently cited article, 53 times in two years, according to Google Scholar) also mentioned that “*The lack of a formal definition has led research to evolve into multiple and inconsistent paths*” and “*It is necessary to invest time and effort in the proposition and the acceptance of a standard definition of Big Data that would pave the way to its systemic evolution and minimize the confusion related to its usage*”. A summary of the definitions used in the Big Data related PubMed Nursing Journals can be found in Appendix Table A2.

Whether the existing definitions are suitable to nursing scholars is also questionable since the definitions were created by data scientists who were interested in developing complicated algorithms to handle Big Data. In contrast, nursing scholars are more interested in analyzing Big Data in order to obtain better research evidence to better inform nursing practice. Nevertheless, the discussion on Big Data in nursing literature is small. When conducting the same keyword search on “Big Data” from all PubMed Journals, almost 2500 publications can be found in addition to the 38 PubMed Nursing Journal publications. Although it is not surprising that significantly higher number of papers about Big Data are published when not restricting to nursing journals, a difference of over 65 times in number is much greater than expected. Out of all, in view of the dynamic nature of the definition for Big Data and the intrinsic needs of nursing and different professions for the advancement in Big Data research, an attempt is made in this paper to discuss the issue using a case study for illustration, and to identify a definition that may be suitable to nursing research.

## 3. A Widespread Definition of Big Data: 4Vs

The definition of Big Data has been described as a “moving target” because there are at least 32 such definitions available from different sources [21,22]. The most popular and widespread of these is the “4Vs” because of its clear structure [19] and because the concept originated from a data scientist [23]. According to this definition, Big Data should fulfill four characteristics that can be represented by four keywords starting with the letter “V”. These characteristics are “Volume”, “Velocity”, “Variety”, and “Veracity”.

The term “Volume” refers to the size of the dataset measured by the unit byte. The second keyword “Velocity” refers to the speed at which new data is being generated. A source of Big Data that generates data at a high speed would naturally produce large volumes of data. Social media, such as Facebook, are examples of such sources of data. Facebook users upload enormous numbers of postings and photographs; with around 1.6 billion active Facebook users in 2016, the speed requirement for Big Data is clearly being fulfilled [24]. The use of social media postings as a source of data has become popular [25,26].

The third keyword “Variety” refers to the number of data types, sources, or attributes. Numeric value is the most popular type of data in a typical nursing study; however, data can also appear in different formats. For example, a database from hospitals can appear in formats other than numeric value, such as images, videos, and text. Image data are generated when patients have X-rays taken in the radiology department, while video data can be generated from gastroscopy examinations in the endoscopy department, and text data are commonly found in patients’ hospital records, generated by physicians, nurses, and other healthcare professionals. Data from the finance department can also be used to conduct health economic studies. Studies that are wider in scope may also be conducted if data from observatories and police departments are used to study the relationship between changes in weather, car accidents, and visits to the accident and emergency department of hospitals. The variety of sources of data is indirectly related to requirements relating to the variety of data attributes. This is because datasets with multiple sources of data are generally expected to have more attributes, and to have a larger capability and more flexibility to answer different research hypotheses. Nevertheless, the variety of data attributes makes Big Data different from traditional small data.

The final keyword “Veracity” refers to the accuracy of a database. Based on the “garbage in garbage out” principle, if a database contains too much inaccurate data, the analysis conducted using this database will generate inaccurate results. In the content of Big Data, a certain degree of inaccuracy is acceptable because the volume of data is much larger than in traditional small datasets, offsetting the potential errors.

Although the 4Vs definition of Big Data is popular, it is still difficult for scholars to judge whether a dataset has fulfilled the 4Vs definition. In practical terms, the definition is also difficult to use. With regard to the term “Volume”, the concept of big is difficult to quantify and it is difficult to come up with an objective threshold value to classify whether a dataset is big or small. The concept depends on the times and on the discipline of research. For example, personal computers were far less common two decades ago and a 3.5 inches floppy disk was the standard medium for transferring and storing data. The storage capacity of a 3.5 inches floppy disk was only 1.44 MB. Nowadays, a USB thumb drive has replaced the floppy disk as the standard medium for managing data. The storage capacity of a typical 64 GB USB thumb drive is 44 thousand times greater than the storage capacity of a 3.5 inches floppy disk. In this connection, the definition of “big” will change from generation to generation, depending on advancements in the technologies available to enable Big Data research. Even if only the current generation of technologies is considered, the concept of big is also related to research disciplines and context. In analyses of data, the unit of size most popular among computer scientists is probably the terabyte, since much of their work is related to handling images and sound tracks. In contrast, quantitative nursing scholars are more familiar with the megabyte, as they normally deal with observational (e.g., questionnaire surveys) or interventional studies. As traditional personal computer hardware and software may not be able to handle data at the gigabyte level or larger, scholars in nursing may encounter a barrier when attempting to analyze the so-called Big Data.

A similar situation of the lack of a benchmark threshold value also exists for the characteristics of “Velocity” and “Variety”. Speed is also affected by advancements in technologies. In the old days, the process of generating data using personal computers normally involved only one party at a time and took place only when people intended to create data, such as data inputs or measurements. The process of generating data is now much more complicated and normally involves multiple parties. Data are not only generated because of deliberate data entries; rather, people’s daily activities indirectly create large amounts of unintentional data. For example, a person’s physical location can be captured by telecommunications companies through the GPS system of that person’s smart phone. Even without any communication device, data can also be generated and captured when people use tap-and-go cards to make small payments. As with the issue of how big is big in terms of “Volume”, it is also difficult for those in different research disciplines to come to a consensus on “Velocity” and “Variety”. Although a social media researcher and a health administration researcher may both claim that they are conducting research using “Big Data”, the former, who would be conducting research using Facebook postings, would have a very different perception of data speed from the latter, who would be conducting research using hospital records, as it is obvious that the speed with which 1.6 billion active Facebook users generate data has to be much faster than that of a hospital or even a hospital network.

The characteristic of “Variety” also presents a challenge for those attempting to come up with a universal standard on how many types of data would be enough to fulfill the criteria of variety. In fact, there are many techniques for analyzing different types of data including texts and images, which require transforming non-numeric types of data back to a numeric value format. For example, linguists may conduct a Big Data study using Facebook posts in text format. They may need to transform the texts to numeric values by counting the number of keywords in the posts in order to conduct statistical comparison tests. Hence, the necessity for Big Data to consist of data in a variety of formats is also debatable. Even shifting the discussion from the number of data formats to data sources, it is also questionable if multiple sources of data are necessary. This is because the number of data sources required should depend on the research hypothesis and on the aims of the study. Having extra sources of data will generally not increase the robustness of a study, except in the case where the data is cross-checked to improve its accuracy. Moreover, having too many attributes could also be troublesome in a data analysis unless the attributes are linearly independent of each other. This is because linearly dependent attributes will not be able to provide additional information as they are directly related to each other [27].

Unlike “Volume”, “Velocity”, and “Variety”, defining the criterion of “Veracity” presents less of a challenge because it has long been an important requirement in data analyses, even before the birth of Big Data. Although it can be difficult to say what level of inaccuracy is considered to be acceptable, it is commonly assumed that the more accurate the data is, the better. Furthermore, this requirement has been relatively stable across different disciplines and generations.

## 4. One Definition of Big Data for Nursing Scholars

It is worth exploring the possibility of having a suitable definition of Big Data for nursing scholars. If, despite the challenges involved, the 4Vs definition of Big Data is adopted in the area of nursing science, nursing scholars may have to face the problem that some of the studies that claim to have adopted Big Data have actually not done so. Moreover, the 4Vs definition may become a barrier to nursing scholars to joining the Big Data research family because of the independent nature of nursing scholars, who are not unique in this regard [6]. In particular, nursing scholars do not typically anticipate themselves to have the relevant computational skills, software, and hardware equipment to handle big data of tremendous volume and at a very fast high speed. It is because these computational skills are expected to be the expertise of computer scientists [6].

In the book “Big Data: A Revolution That Will Transform How We Live, Work and Think” [2], the concept of “sample equals to population” is discussed. There, a more suitable definition of Big Data for nursing scholars is proposed. More criteria could be added to improve the definition, such as by crystallizing the concept of big through imposing an objective threshold, even though that threshold may not perfectly suit all situations. Basically, the concept of “sample equals to population” simply suggests that, for Big Data studies, various sampling processes are no longer required and that the study sample is simply equivalent to the study population in the Big Data world. Thus far, this concept is obviously unfeasible for nursing research. For example, it is not cost-effective to conduct a population census in order to answer a research question for a typical nursing research study. Hence, nursing researchers utilize various sampling methods to select the appropriate subjects to represent the population for research. However, with the world now evolving to enter the data era and with data covering nearly the whole population becoming increasingly available, more opportunities have arisen for nursing scholars to conduct large population-based studies with Big Data. If the concept of “sample equals to population” is adopted as the definition of Big Data in nursing research, it will be easier to classify in a more objective way whether such a nursing study is one that involves Big Data. Conducting Big Data research will become more conceptually clear to nursing scholars. The situation that nursing scholars are excluded from the Big Data world of research is preventable if they can identify a suitable Big Data definition.

## 5. A Case Study of Disaster Preparedness Research Using Big Data

According to the International Council of Nurses (ICN) Framework of Disaster Nursing Competencies, preparedness competence is one of the four major disaster nursing competencies [28]. Although disaster preparedness is considered to be the most critical phase in the disaster management continuum, none of the publications related to disaster preparedness in nursing can be identified in the PubMed Nursing Journals as having used Big Data. Disaster preparedness research using Big Data has, however, been found in the publications of health geographers [5,29], an indication that health geographers are among the first to use Big Data in such research.

An example is a Big Data Analytic study exploring the relationship between changes in weather and the demand for ambulances, which concerns the development of evidence-based practices relating to disaster preparedness in health services and nursing [30]. The study utilized a big dataset of the Hong Kong Hospital Authority (HA—a public hospital system in Hong Kong), which contains six million records of patient visits to the Accident and Emergency Departments (AED) of the hospitals under the HA from May 2006 through April 2009 (3 years). The aim of the study, which was to develop a spatio-temporal daily emergency ambulance demand forecast system, contributes to the knowledge base on the disaster preparedness of emergency ambulances and related health services [30]. If the 4Vs Big Data definition is adopted, since the dataset from the HA can be stored in a compact disk in the format of a single Microsoft Access file of around 500 MB in size, the study would not have been classified as Big Data in terms of the “volume” of the data involved. However, the dataset of the HA contains the total number of AED patient visits with emergency ambulance access in Hong Kong, and covers the whole population of Hong Kong, which meets the criterion for the definition of big data as “sample equals to population” [2]. At the same time, we have to emphasize that the definition of “sample equals to population” is an example to demonstrate the need of a suitable Big Data definition for nursing scholars. In principle, the definition still needs to be improved based on the realistic situation of nursing scholars’ comments and general consensus. For example, some scholars may not agree that the above mentioned dataset is large enough to be called “Big Data”. It is because the dataset with six million records could be considered as big in Hong Kong but small in big country like USA. Moreover, the concept of “Big” could also be changed due to technology advancement such as the change from storing data in floppy disks to USB thump drives. Hence, the concept of Big Data is complicated and clearly depends on both social and technical aspects [31]. Nursing scholars from different specialties should be involved in formulating the Big Data definition for nursing scholars.

The Big Data Analytic study was divided into three phases of data analysis to establish the weather-ambulance demand relationship and the socio-demographic characteristics of users, so as to forecast the short-term and long-term demand for ambulances. In phase one, the daily ambulance demand time-series data by the different socio-demographic characteristics of ambulance users (e.g., age and gender) were extracted to correlate with different meteorological time series data (e.g., temperature and relative humidity) in order to identify the meteorological variables that were significantly correlated with ambulance demand by people’s socio-demographic characteristics. The research results indicated that older people, females, those with a lower income, and patients with more severe conditions were more sensitive to extreme weather conditions [8].

In phase two, a spatio-temporal daily emergency ambulance demand forecast system using the Hong Kong Observatory’s seven-day weather forecast report was developed. The model validation results showed that it is feasible to use a weather forecast report to forecast the future daily demand for ambulances [30]. The accuracy of the forecast was further improved by introducing the seven-day average temperature forecast into the emergency ambulance demand forecast model [29].

In phase three, a long-term projection algorithm to project the demand for ambulances until 2036 was developed, using the corresponding relationship between weather and ambulance demand and the population projection report compiled by the Hong Kong Planning Department. The projection showed that, if the factor of an aging population was neglected, both the yearly demand for ambulances and the sensitivity of the daily demand for ambulances under extreme weather conditions would be severely underestimated [32]. One implication for disaster preparedness during extreme weather conditions that are potentially or actually disastrous is that older people have a higher demand for ambulances. There should be a greater focus on offering disaster preparedness education and relevant health services to this group of citizens.

With the success of this Big Data Analytic study on the relationship between changes in weather and emergency ambulance demand, the same concept and procedures were translated into another study using the health-related help-seeking behavior data of the “personal emergency link” (PE-Link) service for older people. The PE-link service is a telehealth system for older citizens operated by the Senior Citizens Home Safety Association (SCHSA), which has more than 80,000 active users in Hong Kong [33]. The research results also identified a certain group of PE-link users who are relatively sensitive to extreme weather conditions that can be potentially disastrous, e.g., extremely cold days, which are not uncommon during winter in Hong Kong. That study suggested that it is feasible to predict the health-related help-seeking behavior of PE-Link users using Big Data from official government weather forecast reports. The study provided nursing researchers with insights on the benefits of strengthening the use of the PE-link system for disaster preparedness involving older people [34].

The above multi-phased process studies demonstrated that “sample equals to population” Big Data can be analyzed from different perspectives (i.e., the effects of weather on ambulance demand and the effects of population changes on ambulance demand). The use of Big Data has demonstrated its superiority in public health nursing to the sampling approach adopted in traditional nursing research that usually includes only the selected research participants for particular research topics. The data collection process of such traditional research studies is also often time-consuming and demands much effort. The “sample equivalent to population” type of Big Data approach can be used with patient records, which can be analyzed using datasets other than weather reports and can generate results in other aspects that contribute information on public health. In fact, the case study demonstrated that conventional data collection methods (such as questionnaire surveys and in-depth interviews) are not the only ways to conduct disaster preparedness research. When the Big Data approach is used appropriately, more opportunities will be available to nursing scholars to conduct disaster preparedness research because they are no longer limited under the conventional approach. The given example in disaster nursing is only the tip of the iceberg. Other examples of Big Data analytic applications include clinical operations, patient profile analytics, device/remote monitoring, and pre-adjudication fraud analysis. A good review on Big Data analytics in healthcare can be find from Raghupathi [35].

## 6. The Use of Big Data from Disaster Preparedness to Public Health

The above example demonstrated that the Big Data approach can be used in disaster preparedness research, contributing to public health and to research on public health. Big Data research is a good match with public health, as the scope of analysis encompasses the whole population [36]. For example, infectious disease surveillance, which is an important area of public health, was limited in the past by passive methods of collecting data, such as notifiable cases of disease reported by physicians or laboratories [37]. In particular, in the case of influenza, it is likely that the passive reporting approach would have resulted in a significant number of missing cases, due to false negative diagnoses or to the recovery or death of people who had become infected but who had not undergone any medical consultation. If Big Data is adopted in public health surveillance, the reported number of infected cases could be improved. This is because the sources of data will not only be limited to medical records, but also include indirect data such as sales records for flu medicines or records of Internet searches on flu-related subjects, which could be used to estimate the number of infections. In fact, the occurrence of Big Data has helped public health to evolve into public health 2.0, which describes public health activities that are completely user-driven by the use of social media, search engines, mobile technologies, or other technologies [38]. In the new version of public health, the data to conduct infectious disease surveillance are obtained directly from public entities, such as Google Flu Trends and the Global Public Health Intelligence Network [20,39].

## 7. Future Challenges of Using Big Data in Nursing Research

Despite the advantages of conducting nursing research using Big Data, there are some foreseeable challenges that may inhibit its development. The governments of major cities in the world are now emphasizing the use of Big Data to manage cities (e.g., the smart city) and promoting open data policies to increase the public’s involvement in formulating policies. When such ideas are promoted, the issue of data privacy should not be neglected. The public is becoming increasingly concerned about personal privacy. It is projected that it will not be enough to employ data anonymization in connection with the use of Big Data. More rigorous and standardized measures need to be developed to meet the public’s expectations of personal privacy.

In the case of sampling in survey research designs, data anonymization is generally sufficient to assure confidentiality and privacy, as the datasets are specific and the number of research participants is limited. In Big Data analysis, computer algorithms are available to perform data de-anonymization [40]. However, there have been reports showing that merging multi-anonymous Big Data can generate personally identifiable data [1]. For example, assume that there is simple, anonymous Big Data containing all of the records of patients who have visited AEDs in Hong Kong. The dataset contains only six variables (date of visit, time of visit, age, gender, hospital name, and diagnosis). The structure of the data is illustrated in Table 1. If there is a newspaper report about a politician’s visit to an AED, their medical diagnosis can then be easily identified based on the time of the visit, the specific hospital that was visited, and the politician’s gender and age. In this connection, the accessibility of Big Data from both the public and private sectors can be unfavorable to privacy.

In order to have a sustainable environment for encouraging the use of Big Data in nursing research, it is essential to devise a set of standard operating procedures (SOPs) on security measures to ensure privacy and confidentiality when using Big Data for research. For example, researchers can only be allowed to access the data in a dedicated room, all data should be password protected, all data should be destroyed within a certain period after the end of a project, and so on. In fact, the Health Insurance Portability and Accountability Act Privacy Rule has a guideline that includes a list of variables that should be removed from a dataset so that the dataset can be considered to have been de-identified [41]. It is also necessary to invite the public to join in the development of SOPs through open discussion forums.

In fact, the future challenges of using big data in nursing research is not only limited within the issues of privacy and confidentiality. There are many future challenges in Big Data such as (1) deciding the acceptable amount of missing data; (2) addressing the issue of data accuracy and validity; (3) handling large number of confounders; and (4) the impact of using proxy variable. Unlike the issue of privacy and confidentiality which should directly be handled by nursing scholars, other professions such as data scientists and statisticians will play an important role in handling these issues. Hence, there is room for them to multi-disciplinarily collaborate with nursing scholars in formulating a suitable and the best Big Data definition for nursing research in public health.

## 8. Conclusions

Big data is an emerging area of research that could lead to the development of the next generation of more sophisticated and compelling research for evidence-based nursing practices. However, for the time being, in nursing research as in many other disciplines, the existing definitions of Big Data may not be suitable. In this paper, the use and limitations of the 4Vs definition of Big Data for nursing research was introduced and discussed. The suitability of the concept of the whole population as the sample for the definition of Big Data for nursing research was proposed. A case study of using Big Data to conduct disaster preparedness research was also provided to demonstrate that conventional data collection methods (such as sampling surveys and in depth interviews) are not the only ways to conduct disaster preparedness research. Finally, nursing scholars should come to a consensus on the definition of Big Data and establish widely accepted SOPs on security measures to address the issue of the privacy and confidentiality of Big Data, so that research using Big Data in nursing science can be further developed and sustained.

## Figures and Tables

**Table 1:** Part of the simple, anonymous AED Big Data related to the suspected patient.

Date	Time	Gender	Age	Hospital Name	Diagnosis
20 May 2016	14:57	Male	35	A *	Flu
	15:00	Male	60	A	Schizophrenia
	15:05	Female	17	A	Tuberculosis

*: ”A” is a label for the hospital.

## Appendix A

**Table A1:** The 38 Big Data related publications in PubMed Nursing Journals.

No.	Author	Year	Manuscript Title	Journal Name
1	Bakken and Reame [42]	2016	The Promise and Potential Perils of Big Data for Advancing Symptom Management Research in Populations at Risk for Health Disparities	Annual Review of Nursing Research
2	Barton [43]	2016	Big Data	Journal of Nursing Education
3	Blair [44]	2016	Publicly Available Data and Pediatric Mental Health: Leveraging Big Data to Answer Big Questions for Children	Journal of Pediatric Health Care
4	Booth [45]	2016	Informatics and Nursing in a Post-Nursing Informatics World: Future Directions for Nurses in an Automated, Artificially Intelligent, Social-Networked Healthcare Environment	Nursing Leadership
5	Broome [46]	2016	Big Data, Data Science, and Big Contributions	Nursing Outlook
6	Clancy and Reed [15]	2016	Big Data, Big Challenges: Implications for Chief Nurse Executives	Journal of Nursing Administration
7	Linnen [47]	2016	The Promise of Big Data: Improving Patient Safety and Nursing Practice (Part 1)	Nursing
8	Linnen [48]	2016	The Promise of Big Data: Improving Patient Safety and Nursing Practice (Part 2)	Nursing
9	Remus [49]	2016	The Big Data Revolution: Opportunities for Chief Nurse Executives	Nursing Leadership
10	Zurlinden [50]	2016	Health Professions Education: Make Big Data Meaningful	The Journal of Continuing Education in Nursing
11	Brennan and Bakken [14]	2015	Nursing Needs Big Data and Big Data Needs Nursing	Journal of Nursing Scholarship
12	Cohen et al. [51]	2015	Challenges Associated with Using Large Data Sets for Quality Assessment and Research in Clinical Settings	Policy, Politics & Nursing Practice
13	Cohen et al. [52]	2015	Implementing Common Data Elements across Studies to Advance Research	Nursing Outlook
14	Elgin and Bergero [53]	2015	Technology and the Bedside Nurse: An Exploration and Review of Implications for Practice	Nursing Clinics of North America
15	Finfgeld-Connett [54]	2015	Twitter and Health Science Research	Western Journal of Nursing Research
16	Harper and Parkerson [55]	2015	Powering Big Data for Nursing Through Partnership	Nursing Administration Quarterly
17	Henly et al. [56]	2015	Emerging Areas of Science: Recommendations for Nursing Science Education from the Council for the Advancement of Nursing Science Idea Festival	Nursing Outlook
18	Jackson [57]	2015	Teaching and Learning About Big Data: Start Small	Nurse Educator
19	McCartney [58]	2015	Big Data Science	The American Journal of Maternal/Child Nursing
20	McConnell et al. [59]	2015	The Future of Big Data: Innovative Methodological Approaches	Issues in Mental Health Nursing
21	Price et al. [60]	2015	The Veterans Affairs‘s Corporate Data Warehouse: Uses and Implications for Nursing Research and Practice	Nursing Administration Quarterly
22	Samuels et al. [61]	2015	Using the Electronic Health Record in Nursing Research: Challenges and Opportunities	Western Journal of Nursing Research
23	Sensmeier [62]	2015	Big Data and the Future of Nursing Knowledge	Nursing Management
24	Simpson [63]	2015	Big Data and Nursing Knowledge	Nursing Administration Quarterly
25	Stifter et al. [64]	2015	Nurse Continuity and Hospital-Acquired Pressure Ulcers: A Comparative Analysis Using an Electronic Health Record “Big Data“ Set	Nursing Research
26	Stifter et al. [16]	2015	Proposing a New Conceptual Model and an Exemplar Measure Using Health Information: Technology to Examine the Impact of Relational Nurse Continuity on Hospital-Acquired Pressure Ulcers.	Advances in Nursing Science
27	Welton and Harper [65]	2015	Nursing Care Value-Based Financial Models	Nursing Economics
28	Westra et al. [66]	2015	Nursing Knowledge: 2015 Big Data Science	CIN: Computers, Informatics, Nursing
29	Westra et al. [67]	2015	Nursing Knowledge: Big Data Science-Implications for Nurse Leaders	Nursing Administration Quarterly
30	Henly et al. [68]	2014	Mother Lodes And Mining Tools: Big Data For Nursing Science	Nursing Research
31	Lee [17]	2014	A Systems Science Approach to Fatigue Management in Research and Health Care	Nursing Outlook
32	Lyon et al. [18]	2014	Biobehavioral Examination of Fatigue across Populations: Report from a P30 Center of Excellence	Nursing Outlook
33	Maughan et al. [69]	2014	Standardized Data Set for School Health Services: Part 1—Getting to Big Data	NASN School Nurse
34	Rashbass and Peake [70]	2014	The Evolution of Cancer Registration	European Journal of Cancer Care
35	Skiba [71]	2014	The Connected Age: Big Data & Data Visualization	Nursing Education Perspectives
36	Westra and Choromanski [72]	2014	Amazing News for Sharable/Comparable Nursing Data to Support Big Data Science	CIN: Computers, Informatics, Nursing
37	Harper [13]	2013	The Economic Value of Health Care Data	Nursing Administration Quarterly
38	Salcido [73]	2013	Big Data and Disruptive Innovation in Wound Care	Advances in Skin & Wound Care

**Table A2:** The Big Data definition appeared in the 38 Big Data related publications PubMed Nursing Journals.

No.	Big Data Definition	Reference
1	3Vs or 4Vs	[14,42,45,47,48,55,58,60,61]
2	Large pools of data that can be captured, communicated, aggregated, stored, and analyzed, are now part of every sector and function of the global economy	[13]
3	The formation of large electronic repositories of healthcare data	[15]
4	The complexity, challenges, and new opportunities presented by the combined analysis of data. In biomedical research, these data sources include the diverse, complex, disorganized, massive, and multimodal data being generated by researchers, hospitals, and mobile devices around the world.	[43]
5	It is diverse, complex, disorganized and multimodal data generated by hospitals, researchers, and individuals who wear mobile devices and sensors that provide real time data about health status and parameters	[46]
6	The exponential growth of information and knowledge emerging from sources.	[49]
7	Any collection of data that is large and complex enough to become difficult to process.	[52]
8	Techniques developed to manage, analyze, and translate the vast and expanding amount of patient data elements	[53]
9	Data sets that perform beyond the capabilities of traditional software programs to store, manage, and analyze	[57]
10	Datasets whose sizes are beyond the ability of typical database software tools to capture, store, manage, and analyze	[62]
11	Data sets that are of a size or complexity that is beyond the ability of typical database software tools	[64]
12	High volume or complex data that originates from a variety of sources.	[67]
13	The use of ginormous bits or sets of data in the terabyte-and-beyond range, which are exponentially increasing by the minute, owing to the capacity of data collection, storage, and analysis	[73]
